# Biochar Regulates Contrasting Pb^2+^ and Cd^2+^ Transport Regimes Through Water-Dependent Partitioning Transitions Under Unsaturated Conditions

**DOI:** 10.3390/toxics14090787

**Published:** 2026-09-05

**Authors:** Xin Tan, Yilan Li, Lina Xu, Jing Tian, Jiaqi Tan, Jiuli Ruan, Yang He

**Affiliations:** 1 Key Laboratory of Eco-Industry of Ministry of Ecology and Environment, Chinese Research Academy of Environmental Sciences, Beijing 100012, China; 2 School of Environmental Science and Engineering, Southwest Jiaotong University, Chengdu 611756, China

**Keywords:** adsorption kinetic characteristics, biochar, desorption characteristics, isotherms, model fitting, unsaturated conditions

## Abstract

Biochar has been widely applied for immobilization of potentially toxic elements (PTEs) in contaminated soils; however, its effects on metal transport under transient unsaturated conditions remain insufficiently understood. This study investigated the adsorption, desorption, and transport behavior of Pb^2+^ and Cd^2+^ in biochar-amended soils by combining batch experiments, unsaturated conditions column experiments, and transport modeling. The adsorption kinetics, isotherms, and desorption characteristics of Pb^2+^ and Cd^2+^ were evaluated under different biochar levels, and their redistribution during unsaturated infiltration was further analyzed using soil–water-dependent models. Biochar increased the apparent adsorption capacity of both metals, with a stronger response observed for Pb^2+^ than Cd^2+^. The Elovich and two-constant models provided better statistical descriptions of adsorption kinetics than the pseudo-second-order model, indicating that adsorption rate behavior involved heterogeneous and multi-rate processes. Freundlich fitting showed that Pb^2+^ and Cd^2+^ exhibited non-ideal adsorption behavior, while desorption experiments demonstrated a stronger reduction in apparent Pb^2+^ release compared with Cd^2+^ under biochar. Under transient unsaturated infiltration, Pb^2+^ and Cd^2+^ exhibited distinct transport patterns. Pb^2+^ redistribution was adequately described by a linear partitioning relationship, with R^2^ values ranging from 0.988 to 0.990, indicating relatively stable solid–liquid partitioning within the tested conditions. In contrast, Cd^2+^ showed stronger dependence on soil–water conditions, and an empirical nonlinear water-dependent model improved model performance, increasing R^2^ values from 0.533–0.652 for the linear model to 0.903–0.973. The results suggest that biochar effects on potentially toxic element migration under unsaturated conditions are element-specific, involving both apparent sorption enhancement and water-dependent redistribution processes. This study highlights that biochar performance in potentially toxic elements remediation should not be evaluated solely based on equilibrium adsorption capacity. Under field-relevant unsaturated conditions, amendment effectiveness may also depend on interactions between metal-specific retention behavior and soil hydraulic variability. Further studies integrating structural characterization, long-term aging, and reactive transport modeling are required to validate these processes under heterogeneous field conditions.

## 1. Introduction

Potentially toxic element contamination in agricultural soils is a persistent environmental concern because metals can accumulate in soils, enter food chains, and remain difficult to remove once introduced. Regional mapping in Europe showed that at least one potentially toxic element exceeded applied threshold concentrations over 1.2 million km^2^, equivalent to 28.3% of the EU land area, with industrial and mining regions showing elevated As, Cd^2+^, Pb^2+^, and Hg levels [1]. According to the 2014 National Soil Pollution Survey Bulletin, the overall exceedance rate of soil sampling sites nationwide was 16.1%, and that of agricultural (cultivated) land was 19.4%, with samples containing potentially toxic elements or metalloids accounting for the majority of the contaminated samples [2,3]. Subsequently, the Risk Control Standard for Soil Contamination of Agricultural Land (GB 15618-2018), issued in 2018, established a new system of risk screening values and risk intervention values for the risk management of agricultural soil contamination [4]. Meta-analyses further indicate that Cd^2+^ is among the most prevalent pollutants (PTEs) in Chinese farmland soils, with higher potentially toxic element content often reported in the southwest and south coastal regions, as well as in paddy and vegetable fields [2,5,6]. These findings justify continued attention to Cd^2+^ and Pb^2+^ behavior in agricultural soils, particularly in regions affected by mining, industrial inputs, irrigation, and intensive agricultural management.

Pb^2+^ and Cd^2+^ are of particular concern because they show contrasting geochemical behavior in soil–water systems. Potentially toxic element adsorption in soils is controlled by soil type, metal speciation, metal concentration, pH, solid-to-solution ratio, and contact time, and major sorbents include clay minerals, metal (hydr) oxides, and soil organic matter [7]. Pb^2+^ generally has stronger affinity for mineral and organic surfaces and lower mobility, whereas Cd^2+^ is more exchangeable and more sensitive to changes in solution chemistry and soil conditions [7,8]. This contrast is also reflected in crop-related observations, where Cd^2+^ more readily enters plant roots than Pb^2+^ in some agricultural systems, while Pb^2+^ accumulation can be strongly affected by atmospheric deposition and source-specific inputs [9]. Therefore, Pb^2+^ and Cd^2+^ may respond differently to the same amendment and hydrological conditions.

Biochar has been widely investigated as a soil amendment for contaminant management because its physicochemical properties—including surface area, pore-size distribution, ion-exchange capacity, mineral components, and oxygen-containing functional groups—underpin the immobilization of both inorganic and organic contaminants [10,11] Reviews of contaminated soil remediation indicate that biochar may reduce potentially toxic element mobility and bioavailability through sorption, surface complexation, ion exchange, precipitation, and pH-related effects, although the magnitude of remediation depends strongly on biochar properties, soil characteristics, and the target metal [10,12,13]. In addition to chemical retention, biochar can modify soil physical and hydraulic properties. For example, biochar has been reported to reduce soil bulk density by 3–31%, increase porosity by 14–64%, and increase available water by 4–130% across reviewed studies [14]. Biochar-amended compacted soils can also show increased saturated and residual water contents and reduced unsaturated hydraulic conductivity, indicating that biochar may alter both water storage and water movement [15].

Solute transport under unsaturated conditions is strongly affected by pore connectivity, water distribution and mobile-immobile water exchange. Classical studies on macropores emphasized that large continuous pores can produce rapid non-equilibrium water and solute movement that is not fully described by homogeneous Darcy-type flow concepts [16]. More recent unsaturated transport experiments demonstrated a non-monotonic relationship between solute dispersivity and water saturation, with changes in saturation altering velocity heterogeneity, mobile water fraction, and mass exchange between mobile and immobile domains [17]. These findings provide a hydrological basis for interpreting contaminant migration in amended soils, but they do not directly demonstrate biochar-specific Pb^2+^ or Cd^2+^ immobilization mechanisms. Biochar studies on antibiotic transport further suggest that biochar can reduce contaminant discharge by altering soil hydrology and enhancing sorption, and that hydraulic connectivity between macropore and matrix domains can influence biochar-regulated flux redistribution [18,19]. Because these studies focused on organic contaminants, they are used here as conceptual support for hydrology–sorption coupling rather than as direct evidence for Pb^2+^ and Cd^2+^ behavior. In addition, field aging may modify biochar surface chemistry, pore structure, and sorption behavior over time, indicating that short-term amendment effects should not be directly extrapolated to long-term field performance [20,21].

Despite these advances, the effects of biochar on Pb^2+^ and Cd^2+^ behavior under transient unsaturated flow remain insufficiently quantified, particularly when adsorption–desorption behavior and water-dependent transport are evaluated together. Therefore, this study aims to (i) investigate the adsorption kinetics and isotherm behavior of Pb^2+^ and Cd^2+^ under biochar, (ii) evaluate their desorption behavior and transport under non-equilibrium unsaturated flow conditions, and (iii) develop and apply transport models to quantify solute migration behavior under different amendment levels. Particular attention is given to the soil–water ratio as an empirical state variable describing water distribution during unsaturated infiltration and its relationship with Pb^2+^ and Cd^2+^ transport variability.

## 2. Materials and Methods

### 2.1. Materials and Chemicals

The tested soil was collected from farmland in Dongxing District, Neijiang City, Sichuan Province, China (29°36′32.82″ N, 105°14′18.79″ E), under a rice–rapeseed rotation system. The basic physicochemical properties of the soil were as follows: pH, 6.54; total organic carbon (TOC), 3.81 g/kg; total phosphorus (TP), 0.352 g/kg; total nitrogen (TN), 0.582 g/kg; cation exchange capacity (CEC), 18.2 cmol/kg; and sand, silt, and clay contents of 54.42%, 15.61%, and 29.97%, respectively. According to the USDA [22] textural classification, the soil has a sandy clay loam texture, and according to the same classification system, it is classified as an Inceptisol.

**Figure 1:**
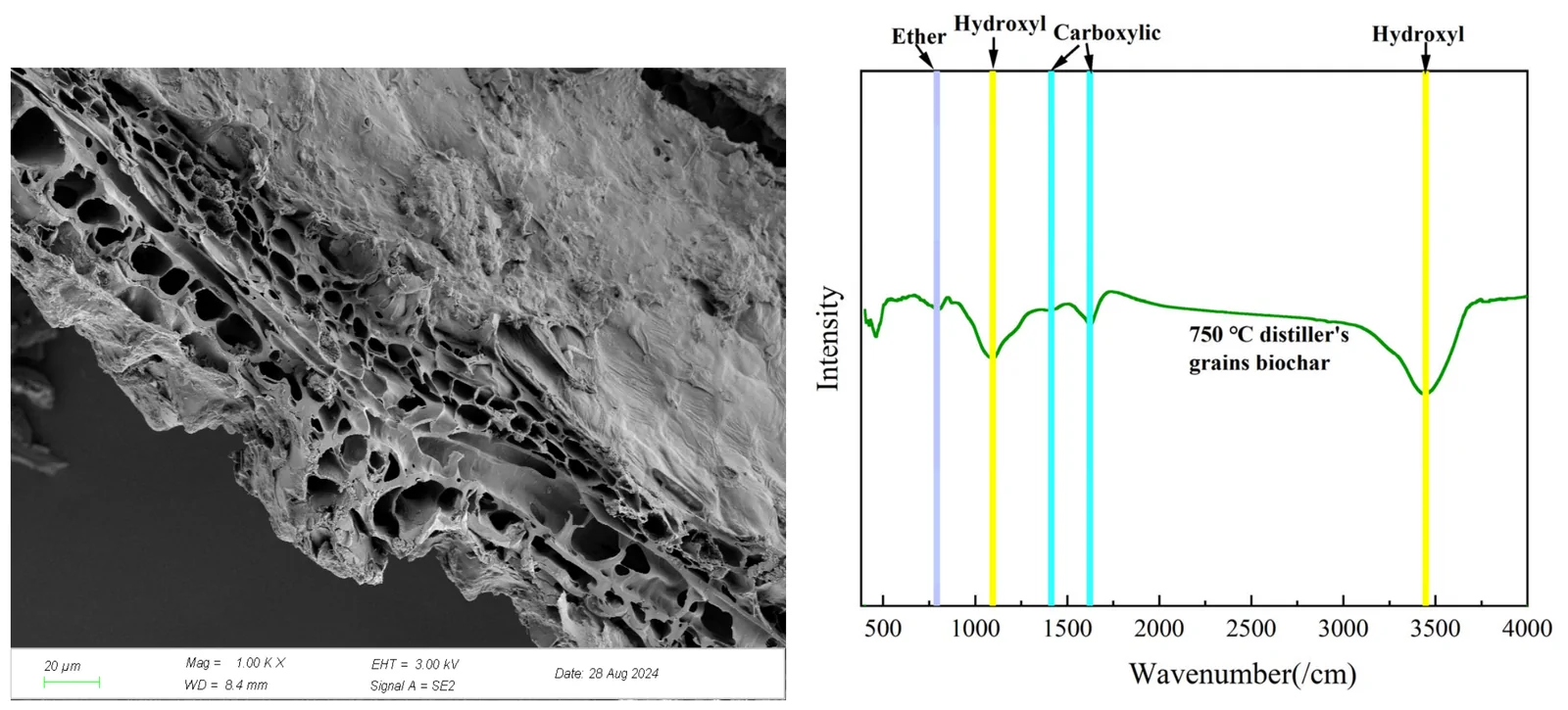
The physicochemical properties of biochar showed SEM morphology analysis of biochar and FTIR functional group analysis. The FTIR spectrum shows the absorbance of the 750 °C distiller’s grains biochar at different wavenumbers.

The biochar was prepared from grains from Baijiu distillers by pyrolysis at 750 °C. It had a carbon content of 81.52% and a specific surface area of 12.83 m^2^/g. The pyrolysis temperature of 750 °C falls within the range of 350–1000 °C defined for biochar production by the European Biochar Foundation [23]. Consistent with this definition, the biochar obtained was a highly carbonized (81.52% C), porous material suitable for contaminant retention. Oxygen and silicon-containing components were detected, and oxygen-containing functional groups, including hydroxyl (-OH) and carboxyl (-COOH) groups, were present on the biochar surface. The FTIR and SEM results of the biochar are shown in Figure 1.

Biochar was added to both uncontaminated and artificially contaminated soils at mass fractions of 1% and 5%. Following previous research, we established a total of six treatment groups in our experiment: control soil (CK), soil amended with 1% biochar (BAS1), soil amended with 5% biochar (BAS5), contaminated soil (CS), contaminated soil amended with 1% biochar (BACS1), and contaminated soil amended with 5% biochar (BACS5) [20].

The contaminated soil was prepared by exogenous addition of Pb^2+^ and Cd^2+^. Briefly, 15.985 g/L Pb(NO_3_)_2_ solution and 0.10975 g/L Cd(NO_3_)_2_ solution were prepared [24], and 200 mL of the mixed solution was gradually and uniformly sprayed onto 4 kg of naturally air-dried and sieved soil. The final Pb^2+^ and Cd^2+^ concentrations in the contaminated soil were 639 mg/kg and 2.3 mg/kg, respectively.

The chemical reagents used in this study are listed in the following Table 1:

### 2.2. Adsorption Experiments

Adsorption kinetics experiment: 1.00 g of tested sample was placed into a 50 mL plastic centrifuge tube. Then, 10 mL of 500 mg/L Pb(NO_3_)_2_ solution or 2 mg/L Cd(NO_3_)_2_ solution was added. The suspensions were shaken at 25 °C and 180 r/min for 5, 30, 240, 360, 1440, 2880, and 4320 min. After shaking, the samples were centrifuged at 4000 r/min for 5 min.

Adsorption isotherm experiment: 1.00 g of tested sample was placed into a 50 mL plastic centrifuge tube. Pb(NO_3_)_2_ solutions at 200, 250, 300, 350, 400, 450, and 500 mg/L, and Cd(NO_3_)_2_ solutions at 2, 4, 6, 8, 10, 12, and 14 mg/L, were added separately. The suspensions were shaken at 25 °C and 180 r/min for 72 h and then centrifuged at 4000 r/min for 5 min.

Desorption experiment: 1.00 g of contaminated soil sample was placed into a 50 mL plastic centrifuge tube, followed by the addition of 10 mL deionized water. The suspensions were shaken at 25 °C and 180 r/min for 0.5, 1.5, 2.5, and 3.5 d and then centrifuged at 4000 r/min for 5 min.

All supernatants were filtered through 0.45 μm membrane filters and analyzed by flame atomic absorption spectrophotometry (model SP-3520AA, Shanghai Spectrum Instruments Co., Ltd., Shanghai, China). All experiments were performed in triplicate.

### 2.3. Unsaturated Column Experiment

**Figure 2:**
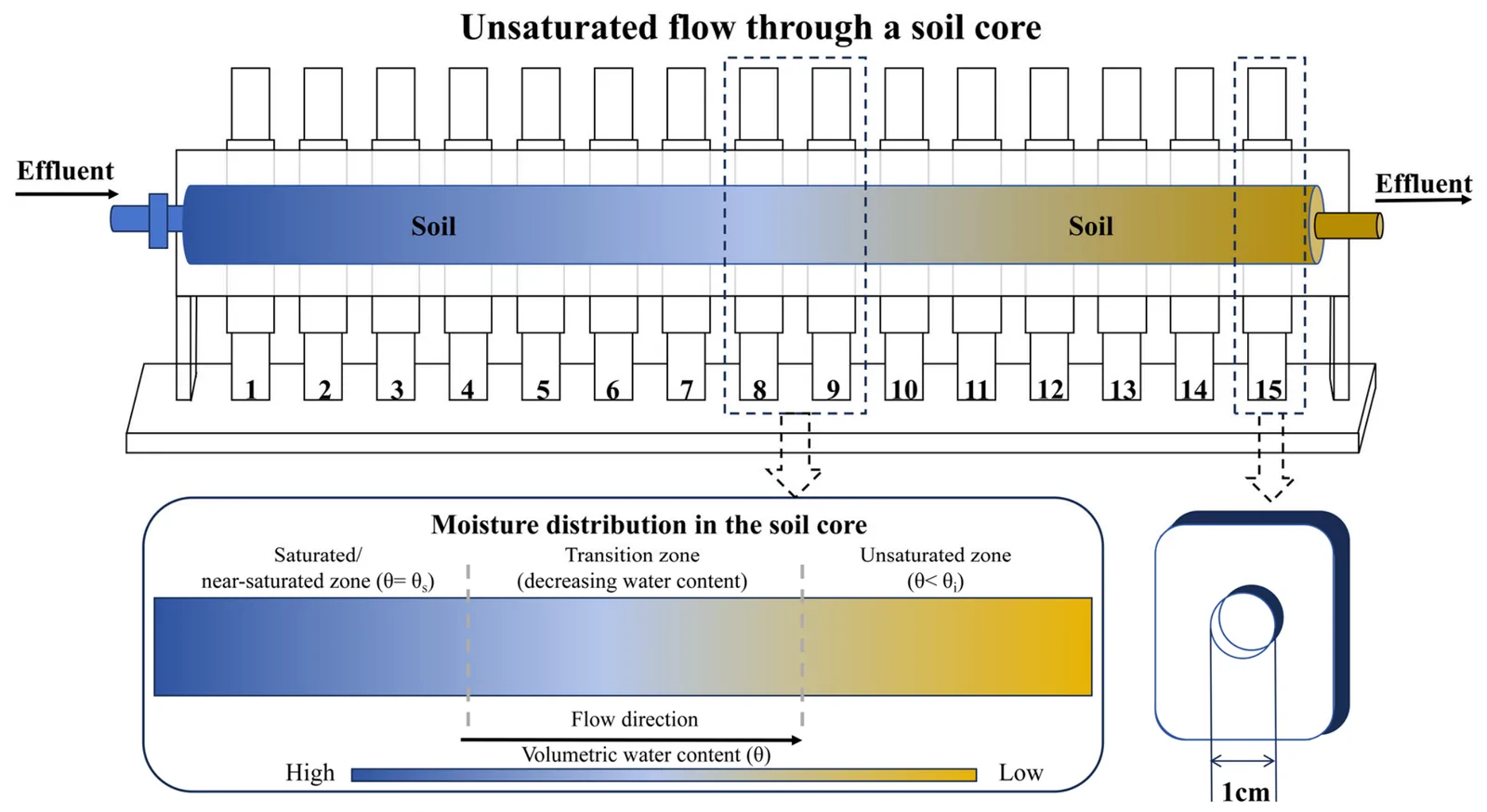
Schematic diagram of the unsaturated transport experiment.

As shown in Figure 2, to construct a multi-layer polypropylene soil column for water infiltration experiments under unsaturated conditions, fifteen resin plates were assembled sequentially, each containing a 1 cm diameter hole for soil packing [19]. The mass of contaminated soil required for packing was calculated according to the designed bulk density, evenly divided into 15 portions, and packed sequentially into the layered compartments to ensure a comparable soil mass in each layer.

Deionized water was allowed to infiltrate the contaminated soil column under gravity, and the advance of the wetting front was monitored during infiltration. When the wetting front reached 13 cm, water supply was stopped, and the column was immediately disassembled layer by layer along the resin plates. Soil samples from each layer were collected for water content and DTPA-extractable Pb^2+^ and Cd^2+^ determination.

Soil water content was determined by oven-drying at 105 °C. The oven-dried samples were extracted with DTPA extractant at a soil-to-solution ratio of 1:2 (g/mL), shaken at 20 °C and 180 r/min for 2 h, and filtered through 0.45 μm membrane filters. Pb^2+^ and Cd^2+^ concentrations in the filtrates were determined by flame atomic absorption spectrophotometry.

### 2.4. Modelling

The adsorption kinetic data were fitted using the pseudo-second-order, Elovich, and two-constant kinetic models. The adsorption kinetic data were fitted using the pseudo-second-order, Elovich, and two-constant kinetic models. The rationale for selecting these models is as follows. The pseudo-second-order model assumes that the adsorption rate is controlled by the active sites on the adsorbent surface and is therefore suitable for describing adsorption processes dominated by chemisorption; the Elovich model is applicable to adsorption on heterogeneous surfaces where the activation energy changes with surface coverage and the adsorption rate decreases with time; and the two-constant model is an empirical model widely used to describe the adsorption kinetics of potentially toxic elements in complex media such as soils. As the three models emphasize different aspects, chemisorption-dominated adsorption, adsorption on heterogeneous surfaces, and empirical description of complex media—their combined use allowed us to examine the adsorption rate behavior from multiple perspectives and to avoid over-interpreting the fit of any single model. Following recent reviews of adsorption kinetic modeling, model fitting results were interpreted as statistical descriptions of adsorption rate behavior rather than direct proof of a unique sorption mechanism [25,26].

The kinetic models are expressed as follows:

Pseudo-second-order kinetic model (PSOM):

(1) $$\frac{t}{q_{t}}=\frac{1}{K_{2}q_{e}^{2}}+\frac{t}{q_{e}}$$

Elovich model:

(2) $$q_{t}=\frac{1}{\beta_{e}}\ln\left(\alpha_{e}\beta_{e}\right)+\frac{1}{\beta_{e}}\ln t=A+B\ln t$$

Two-constant model:

(3) $$q_{t}=e^{\left(a+K_{s}\ln t\right)}$$

where *q_e_* is the adsorption capacity at equilibrium (mg/kg); *q_t_* is the adsorption capacity at time *t* (mg/kg); *t* is adsorption time (min); *K*_2_ is the pseudo-second-order kinetic rate constant; *A* and *B* are Elovich fitting parameters; and a and *K_s_* are two-constant model parameters.

The adsorption isotherm data were fitted using the Freundlich and Henry models. The Freundlich model is an empirical equation suitable for heterogeneous surfaces with non-uniform energy distribution, with the parameter *1/n* reflecting the adsorption intensity and surface heterogeneity; the Henry model is a linear partitioning model used to examine the linearity of adsorption at low concentrations and to serve as a reference for the nonlinear models. Considering that the soil–biochar system investigated in this study possesses a heterogeneous, multisite surface [7,27], a single ideal homogeneous model cannot adequately describe its adsorption behavior; the above combination of models was therefore adopted to characterize the adsorption properties from different perspectives, and all model parameters were obtained by nonlinear regression.

Freundlich model:

(4) $$q_{e}=K_{f}C_{e}^{\frac{1}{n}}$$

Henry model:

(5) $$q_{e}=K_{H}C_{e}$$

where *K_f_* is the Freundlich adsorption coefficient, (mg/kg)·(L/mg)^1/*n*^; 1/*n* is the adsorption intensity parameter, which is temperature-dependent; and *K_H_* is the linear partition coefficient, L/kg.

Solute transport model.

For the solute transport model, this study employed a mass-balance-based analytical framework to describe water and solute movement during unsaturated infiltration in the soil column. In this water-driven displacement framework, infiltrating water is assumed to displace the antecedent soil water, and the position of the water-separation plane is estimated from the water balance. The framework was chosen mainly because it is derived directly from the principle of mass conservation, has clear physical meaning, and requires few empirical parameters, making it highly compatible with the horizontally packed, layered column design of this study. In addition, the column was sectioned into 15 layers for separate measurements of water content and solute content, which correspond directly to the parameters in the framework and allow the underlying assumptions to be tested. Under the assumptions of ideal displacement and adsorption equilibrium, the displacement of the pre-existing soil water by the infiltrating water can be expressed analytically, avoiding the additional calibration of hydraulic parameters required by numerical models. This framework is also consistent with classical unsaturated-flow and solute-transport theory, in which water content, pore connectivity and mobile–immobile water partitioning can affect solute spreading and non-equilibrium transport, matching the unsaturated infiltration conditions of this experiment [16,17,28].

The transient unsaturated water flow model was used to describe solute redistribution during unsaturated infiltration. In this method, water is allowed to infiltrate into a horizontally packed soil column. The original soil water is displaced forward by the infiltrating water and accumulates in the region beyond the water-separation plane, x*. The position of the water-separation plane is calculated as follows:

(6) $$\int_{\theta_{n}}^{\theta_{s}}xd\theta =\int_{0}^{x^{*}}\theta dx$$

where *θ_s_* is the volumetric water content near the inlet of the soil column; *θ_n_* is the volumetric water content at the distal end of the soil column; and x* is the position of the water-separation plane.

If the displacement of the invading water is ideal and solute diffusion is negligible, the soil solution in the region of *x* > x* originates entirely from the pre-existing soil water displaced during infiltration. This assumption is central to the method. If adsorption equilibrium has been reached before water infiltration, the solution composition in the region of *x* > x* should remain unchanged during transport and should be consistent with that of the displaced antecedent solution. Accordingly, the amount of solute adsorbed by the soil in this region should remain constant. Therefore, any variation in solute content within the region of *x* > x* is attributed solely to changes in water content caused by the accumulation of antecedent soil water. In this region, the relationship between the solute content of reactive solute *i*, *M_i_*, and the solution volume per unit soil mass, *θ/ρ*, should be linear:

(7) $$M_{i}=Q_{in}+(\frac{\theta }{\rho })C_{in}$$

where *M_i_* is the solute content per unit mass of soil, mg/kg; *Q_in_* is the adsorbed potentially toxic elements concentration in soil, mg/kg; *C_in_* is the solute concentration in the solution phase, mg/L; *θ* is the volumetric water content, m^3^/m^3^; and *ρ* is the soil bulk density, kg/m^3^.

Because the infiltration process was slow, the adsorption-partitioning process was assumed to reach equilibrium. Therefore, the linear partition model assumes equilibrium partitioning between solid and liquid phases, as commonly applied in soil sorption systems [8].

(8) $$M_{i}=KC_{in}+(\frac{\theta }{\rho })C_{in}$$

The values of *M_i_* and *θ/ρ* in each soil layer were obtained experimentally. Linear fitting of these data was used to estimate the amount of solute adsorbed by the soil and the residual solute concentration in the solution phase at equilibrium before water infiltration, thereby determining the partitioning of solutes between the solid and liquid phases.

All data were processed using Excel 2021. Statistical significance was analyzed using SPSS 27.0 (IBM SPSS Statistics 27), and figures were plotted using Origin 2024.

## 3. Results and Discussion

### 3.1. Adsorption Kinetics

**Figure 3:**
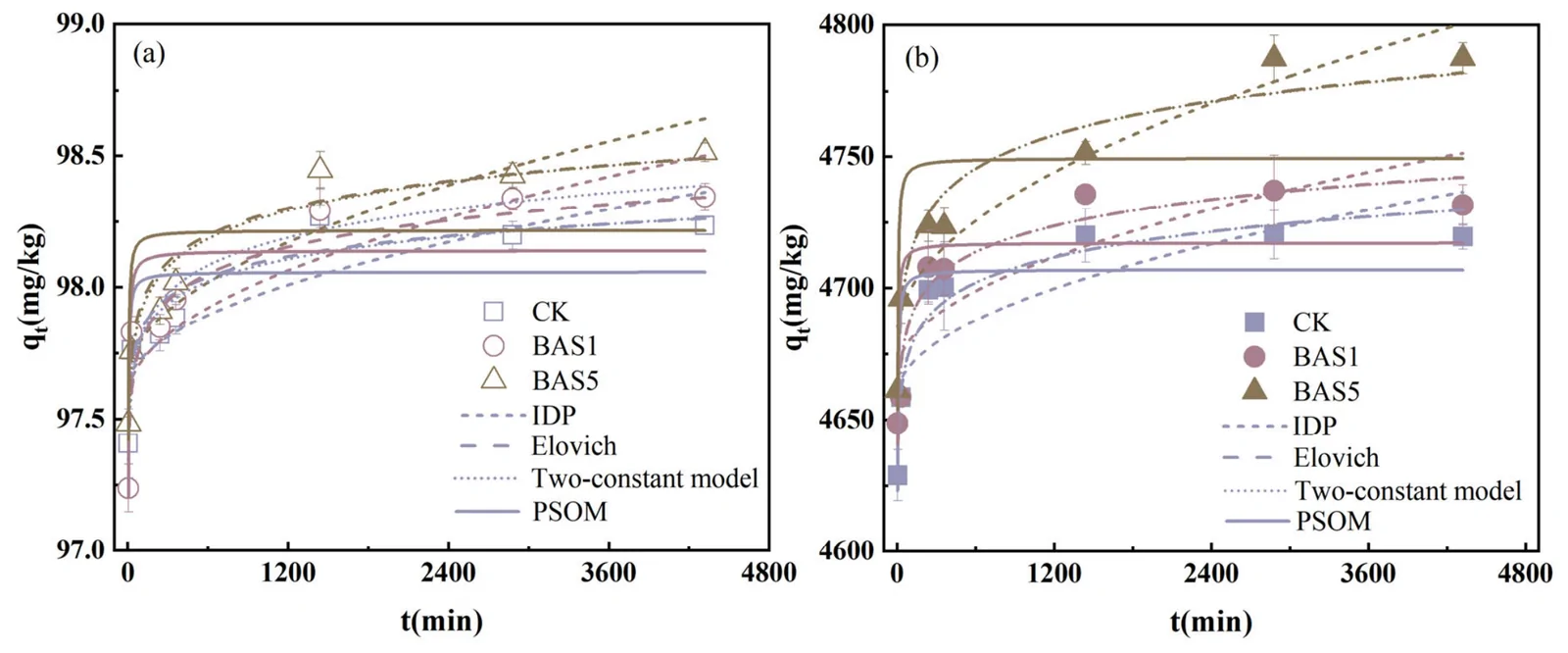
The model fitting of the Cd^2+^ and Pb^2+^ in the adsorption kinetics process showed that the model fitting effect of Cd^2+^ (**a**) and the model fitting effect of Pb^2+^ (**b**) under soil amended with 1% biochar (BAS1), soil amended with 5% biochar (BAS5) and control soil (CK), The models are Elovich model, two-constant model, PSOM, and intra-particle diffusion model (IPD).

Adsorption processes exhibited a two-stage kinetic pattern, characterized by an initial rapid uptake stage followed by a slower stabilization stage. This behavior is commonly observed in heterogeneous sorption systems, where rapidly accessible sorption interfaces are occupied first and subsequent adsorption is progressively limited by slower mass transfer and diffusion processes [25,27]. It should be noted that the kinetic models used in this study are empirical in nature, and their fitting results mainly provide statistical descriptions of the adsorption rate behavior rather than unique mechanistic evidence. As shown in Figure 3, approximately 85%–95% of total adsorption occurred within the first 0–120 min across all treatments, indicating that the early adsorption stage was dominated by rapid solute removal from solution. The subsequent slower stage likely reflects the gradual approach toward adsorption equilibrium and reduced availability of readily accessible sorption interfaces.

At equilibrium (4800 min), Pb^2+^ adsorption increased from 4707 mg/kg in CK to approximately 4765–4780 mg/kg in BAS5, while Cd^2+^ increased from 98.06 mg/kg to approximately 98.5 mg/kg. Although the absolute increase was relatively limited, the higher response of Pb^2+^ compared with Cd^2+^ suggests that it produced element-dependent changes in apparent adsorption capacity under the tested conditions. Previous studies have shown that biochar can enhance PTE retention through a combination of surface functional groups, mineral components, ion exchange capacity, and porous structures. These mechanisms generally act in combination rather than in isolation, and their relative contributions depend on the physicochemical properties of the biochar [10,11,12,13]. However, the specific contribution of individual functional groups or mineral phases cannot be determined from kinetic experiments alone.

As summarized in Table 2, the Elovich and two-constant models generally provided better statistical descriptions of Cd^2+^ and Pb^2+^ adsorption behavior than the pseudo-second-order (PSO) model. The R^2^ values of the Elovich model ranged from 0.922–0.952 for Cd^2+^ and 0.948–0.959 for Pb^2+^, whereas the PSO model showed lower R^2^ values (0.554–0.764). The two-constant model further improved the fitting performance, with R^2^ values of 0.871–0.983 for Cd^2+^ and 0.948–0.960 for Pb^2+^. These results indicate that adsorption rate behavior was not fully represented by a single-rate model and was likely associated with multiple adsorption environments and rate-limiting processes. It should be noted that kinetic model fitting provides a statistical representation of adsorption behavior rather than direct identification of a unique molecular mechanism [26].

Meanwhile, to further elucidate the rate-limiting step of adsorption, the kinetic data were fitted with the intra-particle diffusion model (q_t_ = K·t^0.5^ + C). The fitted intercepts C were significantly greater than zero (4658.71–4681.11 mg/kg for Pb^2+^ and 97.58–97.65 mg/kg for Cd^2+^) and close to the corresponding equilibrium adsorption capacities, indicating that adsorption was nearly completed at the initial stage and that boundary-layer (film) diffusion and surface adsorption contributed predominantly, whereas intra-particle diffusion was not the main rate-limiting step. Moreover, the goodness of fit of the intra-particle diffusion model (R^2^ = 0.667–0.914) was lower than that of the Elovich and two-constant models, further supporting that chemisorption and surface heterogeneity governed the adsorption process.

Parameter analysis further showed that Cd^2+^ exhibited relatively limited variation (<5%) among treatments, suggesting a comparatively stable apparent adsorption response to biochar addition. In contrast, Pb^2+^ under BAS5 showed increases in the Elovich B value (14.01 to 18.26, +30.2%) and the two-constant K_s_ parameter (2.99 × 10^−3^ to 3.89 × 10^−3^, +33.8%). These changes suggest that higher biochar altered the apparent adsorption rate behavior of Pb^2+^, although the underlying surface-level processes require further characterization.

Overall, both Pb^2+^ and Cd^2+^ followed a rapid uptake–slow stabilization kinetic pattern, but their responses to biochar differed in magnitude. Pb^2+^ showed a stronger apparent adsorption response, whereas Cd^2+^ exhibited comparatively limited variation. This difference is consistent with the generally stronger retention tendency of Pb^2+^ in soil systems compared with Cd^2+^, which is more sensitive to solution chemistry and exchange processes [5,6]. Meanwhile, taken together, the model fitting results suggest that the interaction between biochar and metal ions may be dominated by chemisorption: the functional-group complexation sites and surface heterogeneity provided by biochar contributed mainly to Pb^2+^ adsorption (as reflected by the significantly increased Elovich and two-constant parameters under BAS5) [29], whereas Cd^2+^ adsorption may be more closely associated with solution chemistry and ion-exchange processes [11].

### 3.2. Sorption Isotherm

**Figure 4:**
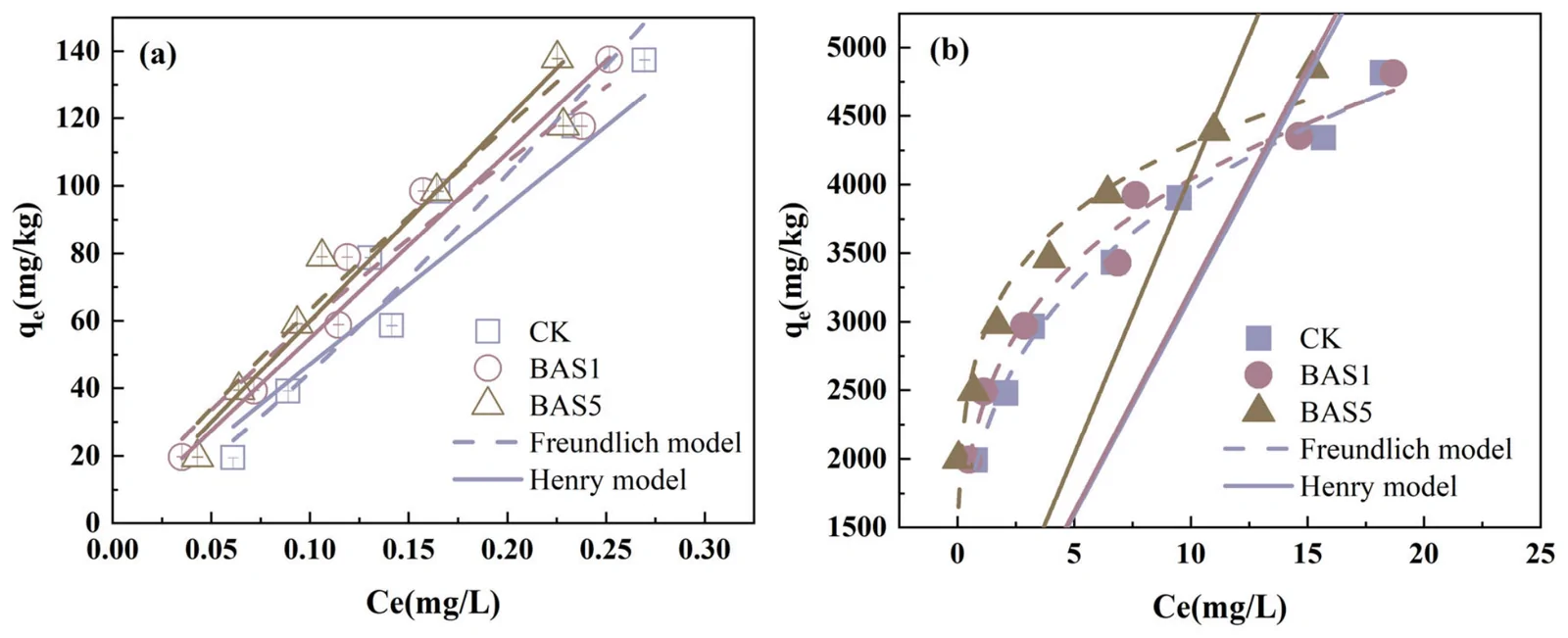
At 25 °C, the adsorption isotherm processes of the Cd^2+^ and Pb^2+^ are shown for the adsorption isotherm models of Cd^2+^ (**a**) and Pb^2+^ (**b**) under the conditions of soil amended with 1% biochar (BAS1), soil amended with 5% biochar (BAS5), and control soil (CK), with the models being the Freundlich model and the Henry model.

As shown in Figure 4, adsorption isotherms of Cd^2+^ and Pb^2+^ in CK, BAS1 and BAS5 treatments exhibited nonlinear increasing trends with increasing equilibrium concentration. Biochar increased adsorption capacity, and BAS5 generally showed higher adsorption than BAS1 and CK. In the high Ce region, q_e_ increased by approximately 10%–20% for Pb^2+^ and 2%–8% for Cd^2+^, indicating that biochar enhanced the apparent partitioning capacity under equilibrium conditions. This enhancement is consistent with previous studies showing that biochar can provide additional sorption interfaces and modify soil–solute interactions through its physicochemical properties [8,10]. In this study, the adsorption isotherms were fitted using the Freundlich and Henry isotherm models; it should be noted that both models are empirical in nature, and their fitted parameters mainly provide statistical descriptions of the adsorption capacity and affinity rather than uniquely determining the underlying adsorption mechanism.

As summarized in Table 3, the Freundlich model provided better fitting performance than the Henry model for both Cd^2+^ and Pb^2+^, with R^2^ values exceeding 0.949 across all treatments. This indicates that adsorption behavior deviated from ideal linear partitioning within the tested concentration range. The Freundlich model describes non-ideal adsorption behavior through an empirical relationship and does not independently prove a specific adsorption mechanism [26].

The Freundlich parameters showed different responses between Pb^2+^ and Cd^2+^. Pb^2+^ K_f_ under BAS5 partially recovered relative to BAS1 (2848.15 to 2339.15 (mg/kg) (L/mg)*^1/n^*), whereas Cd^2+^ exhibited a non-monotonic trend (728.18 → 413.78 → 496.39). Such non-monotonic responses indicate that biochar dosage did not produce a simple linear increase in adsorption capacity. Similar dose-dependent variations have been reported in biochar-amended soils, where amendment level, soil properties, and metal species jointly determine the final adsorption response [10,13]. However, batch isotherm experiments represent equilibrium conditions and cannot fully resolve transient redistribution processes occurring during water flow.

The *1/n* values further indicated different adsorption intensity patterns between metals. Cd^2+^ changed from >1 (1.21) to <1 (0.84–0.90), indicating weaker adsorption intensity and stronger concentration dependence, with its adsorption possibly mainly associated with ion exchange and weak surface complexation [11]; whereas Pb^2+^ maintained relatively low values (0.18–0.28), which may indicate high-affinity adsorption onto heterogeneous surface sites on the biochar surface, consistent with chemisorption processes such as functional-group complexation and surface precipitation [29]. These results suggest stronger apparent adsorption intensity for Pb^2+^ according to Freundlich parameter estimation, while Cd^2+^ exhibited greater concentration dependence under the tested conditions. The difference is consistent with the contrasting soil chemical behavior of Pb^2+^ and Cd^2+^ reported in previous studies [7,8].

Therefore, biochar-induced changes in Cd^2+^ and Pb^2+^ adsorption should be interpreted as element-dependent modifications of apparent sorption behavior rather than a universal enhancement of retention capacity. The observed differences provide a basis for understanding subsequent desorption and transport behavior, although molecular-scale mechanisms require additional spectroscopic or mineralogical evidence.

### 3.3. Desorption

**Figure 5:**
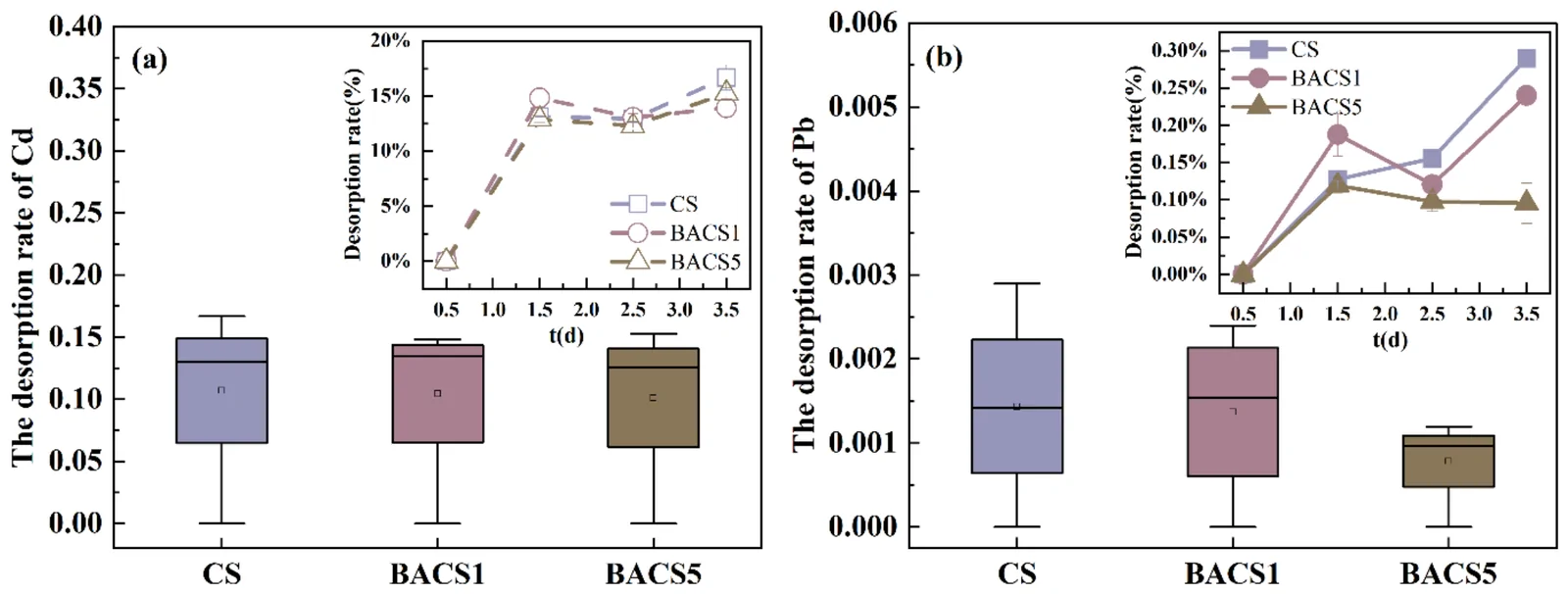
During the desorption process of potentially toxic elements Cd^2+^ and Pb^2+^, the addition of contaminated soil amended with 1% biochar (BACS1), contaminated soil amended with 5% biochar (BACS5), and contaminated soil (CS) shows the comparison of desorption rate of contaminated Cd^2+^ (**a**) and pb^2+^ (**b**) between different biochar addition groups. The small figure shows the trend of potentially toxic elements desorption rate.

As shown in Figure 5, both Pb^2+^ and Cd^2+^ exhibited relatively low desorption intensities across all treatments, followed by gradual stabilization over time. Pb^2+^ desorption reached up to 7.41 mg/kg in CS and decreased to approximately 2.44–3.05 mg/kg under BACS5. In contrast, Cd^2+^ remained at consistently low release levels (≤1.53 mg/kg) across all treatments, with limited temporal variability.

For Pb^2+^, desorption followed the order CS > BACS1 > BACS5 throughout the experiment. BACS5 consistently exhibited the lowest Pb^2+^ release, with a maximum reduction of approximately 67.1% at 3.5 d compared with CS. This indicates that biochar reduced apparent Pb^2+^ release under the tested desorption conditions. For Cd^2+^, treatment differences were comparatively smaller (within 10%–20%), suggesting that Cd^2+^ desorption was less sensitive to biochar addition during the experimental period.

Previous studies have reported that biochar can reduce potentially toxic elements mobility through enhanced sorption capacity, increased ion-exchange sites, mineral interactions and changes in soil chemical conditions [8,10,18]. The stronger reduction in Pb^2+^ desorption observed here is consistent with the generally stronger retention tendency of Pb^2+^ compared with Cd^2+^ in soils [7,8].

Considering the adsorption results together, Pb^2+^ showed stronger apparent retention and lower desorption reversibility than Cd^2+^ under the tested conditions. However, this interpretation is based on macroscopic adsorption and desorption measurements and does not directly resolve molecular-scale binding configurations. Overall, biochar reduced the apparent mobility of both metals, with a more pronounced effect observed for Pb^2+^. The long-term stability of this immobilization behavior requires further evaluation under dynamic field conditions involving water fluctuation, aging, and competing soil components.

### 3.4. Unsaturated Transport

Unsaturated solute transport differs from saturated transport because water distribution, pore connectivity, and mobile–immobile water exchange jointly determine solute movement. In structured soils, macropores and preferential flow pathways may cause non-equilibrium transport that cannot be fully described by homogeneous flow assumptions [16,30]. Recent experimental studies have further demonstrated that changes in water saturation can modify dispersivity, velocity heterogeneity, and the proportion of mobile water domains, resulting in variable solute transport behavior under unsaturated conditions [17]. Therefore, potentially toxic element migration under unsaturated conditions should be interpreted as a coupled process involving aqueous redistribution and solid-phase retention rather than a simple concentration-gradient-driven process.

**Figure 6:**
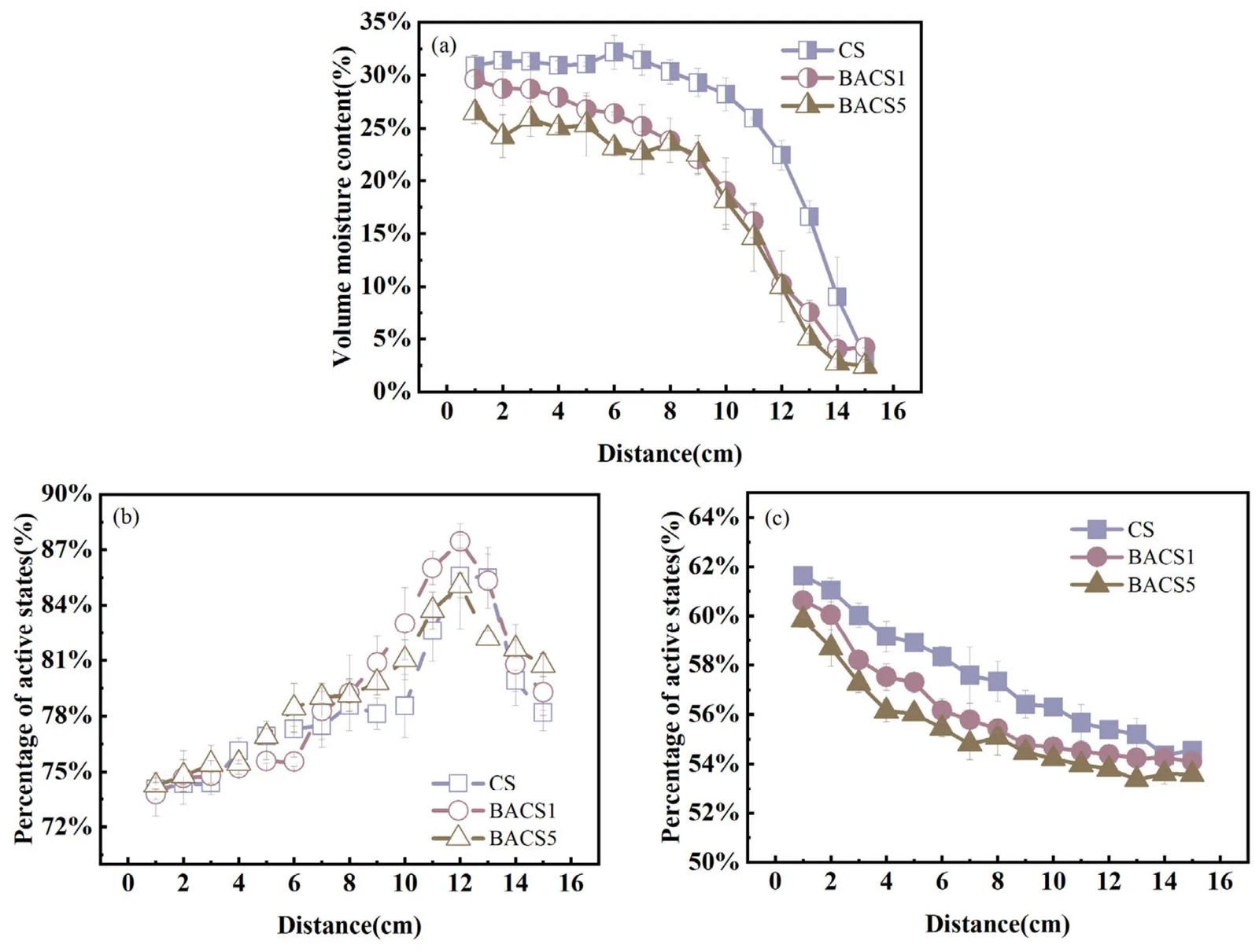
Depth profiles of (**a**) volumetric water content and (**b**,**c**) active fractions of Cd and Pb along the soil column (0–15 cm) under unsaturated conditions for three treatments: CS (contaminated soil without biochar), BACS1 (contaminated soil amended with 1% biochar, *w*/*w*), and BACS5 (contaminated soil amended with 5% biochar, *w*/*w*). (**b**) Active Cd fraction; (**c**) active Pb fraction.

As shown in Figure 6, biochar altered the spatial distribution of Pb^2+^ and Cd^2+^ during unsaturated infiltration. The observed changes should be interpreted as modifications of apparent retention and water-dependent redistribution under the tested conditions. Although biochar may influence soil pore structure and water retention, the present column experiment does not directly resolve pore-scale structural changes. Therefore, the contribution of hydraulic modification is inferred from transport behavior rather than directly quantified.

Pb^2+^ exhibited relatively limited redistribution during unsaturated infiltration compared with Cd^2+^. This behavior is consistent with the generally stronger retention tendency of Pb^2+^ in soils, where mineral surfaces, metal oxides, and organic matter can provide important sorption environments [7,8]. Biochar further reduced the apparent Pb^2+^ mobility, which may be associated with enhanced solid-phase retention capacity through additional reactive interfaces, ion exchange capacity, and mineral-associated interactions reported for biochar-amended soils [10,12,13]. However, the relative contribution of surface complexation, precipitation, ion exchange, or mineral interactions cannot be separated without complementary spectroscopic or mineralogical analyses.

Compared with Pb^2+^, Cd^2+^ exhibited stronger sensitivity to soil–water conditions during unsaturated transport. This difference is consistent with the weaker retention and greater exchangeability of Cd^2+^ in soils, which increases its dependence on aqueous-phase redistribution and solution chemistry [7,8]. The observed relationship between Cd^2+^ distribution and soil–water ratio suggests that Cd^2+^ migration was more responsive to changes in water availability and transport pathways under the tested infiltration conditions. However, this water-dependent relationship is interpreted as an empirical response pattern rather than direct evidence of a pore-scale transport mechanism.

The differential responses of Pb^2+^ and Cd^2+^ highlight that biochar effects cannot be evaluated solely from equilibrium adsorption capacity. Biochar can simultaneously modify reactive interfaces and soil hydraulic properties. Previous studies have shown that biochar can increase water retention, alter pore-size distribution, and modify unsaturated hydraulic conductivity [14,15]. Recent studies on potentially toxic element removal by biochar have demonstrated that enhanced sorption and hydrological changes can jointly influence the migration of potentially toxic elements [29,31], providing evidence directly relevant to the hydrology–sorption coupling for Pb^2+^ and Cd^2+^.

### 3.5. Modeling

**Figure 7:**
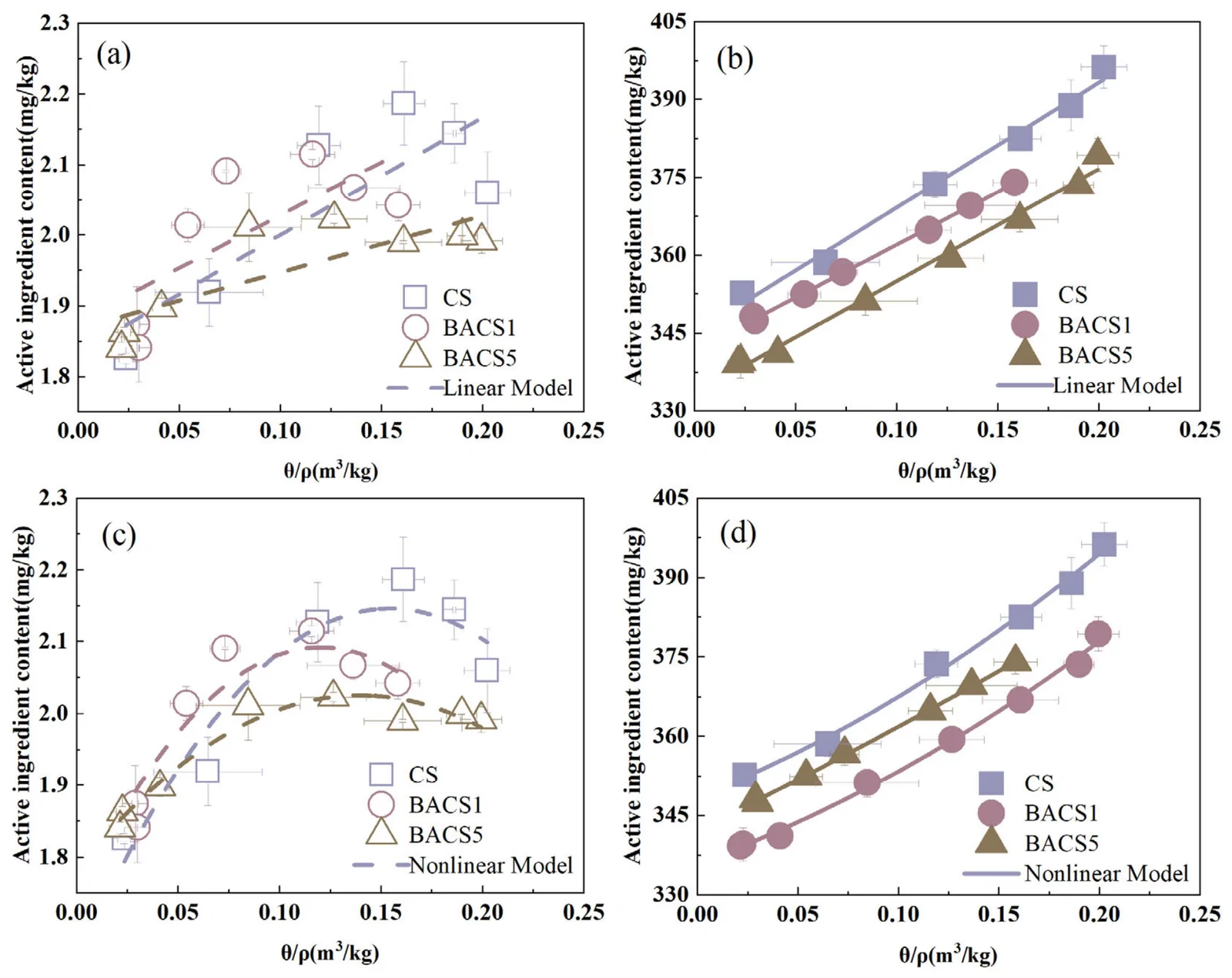
Under the influence of unsaturated transient flow, the active state contents of Cd^2+^ (**a**,**c**) and Pb^2+^ (**b**,**d**) in contaminated soil with 1% biochar (BACS1), contaminated soil with 5% biochar (BACS5) and untreated contaminated soil (CS) were fitted by linear model and nonlinear model.

As shown in Figure 7, The relationship between solute mass and soil–water conditions was evaluated to determine suitable mathematical representations of Pb^2+^ and Cd^2+^ redistribution under transient unsaturated infiltration. Traditional partitioning models assume that solute distribution between solid and liquid phases can be represented by a constant partition coefficient. However, under unsaturated conditions, variations in water content, flow pathways, and mobile–immobile water exchange may influence apparent solute distribution [17,28]. It should be noted that the solute transport model adopted in this study also has limitations: it is established on the assumptions of ideal displacement and adsorption equilibrium and does not explicitly account for diffusion and non-equilibrium sorption, and thus may not fully capture the complex transport behavior under unsaturated conditions. Therefore, alternative model structures were compared to describe the observed transport behavior.

As summarized in Table 4, for Pb^2+^, the linear partitioning model provided a consistently high fitting performance, with R^2^ values ranging from 0.988 to 0.990. The results indicate that a linear representation adequately described Pb^2+^ distribution between solid and aqueous phases within the experimental range. This finding is consistent with the stronger retention tendency of Pb^2+^ in soil systems. However, the success of linear fitting does not imply that Pb^2+^ adsorption is completely homogeneous or irreversible, because potential kinetic effects and microscale heterogeneity may still exist beyond the investigated conditions.

For Cd^2+^, the linear model showed lower performance, with R^2^ values ranging from 0.533 to 0.652. The nonlinear water-dependent formulation improved the statistical description of Cd^2+^ behavior, increasing R^2^ values to 0.903–0.973 with reduced RMSE and AICC values. The improved performance indicates that Cd^2+^ redistribution was better represented when soil–water conditions were included as an explanatory variable.

The empirical nonlinear relationship was expressed as:

(9) $$C_{in}=S+w(\frac{\theta }{\rho })$$

where *w* represents an empirical parameter describing the sensitivity of solution concentration to soil–water ratio and *S* represents a background term. The derived θ/ρ* is obtained by solving Equation (9) using the fitted parameters *S* and *w* with the influent concentration (*C_in_*) set to zero. This formulation should be interpreted as a statistical representation of observed variability rather than a mechanistic transport equation. The nonlinear response may reflect combined effects of water redistribution, solute accessibility, and apparent partitioning changes during infiltration, but these processes cannot be individually separated based only on column-scale observations.

The contrasting model performance between Pb^2+^ and Cd^2+^ suggests that the two metals exhibited distinct transport representations under the tested unsaturated conditions. Pb^2+^ transport was adequately described by a linear partitioning relationship, whereas Cd^2+^ showed a stronger dependence on soil–water conditions and was better represented by an empirical nonlinear formulation. These differences indicate that metal-specific transport behavior cannot be fully evaluated based solely on equilibrium adsorption capacity, particularly under transient unsaturated conditions where water redistribution influences solute accessibility and phase partitioning.

For Pb^2+^, the linear model showed consistently high performance, indicating that the observed Pb^2+^ distribution could be effectively described by an apparent partitioning relationship within the investigated range. The estimated distribution coefficient (K_d_) increased from 1.43 (CK) to 1.68 (BACS1) and slightly decreased to 1.54 (BACS5). This pattern suggests that biochar enhanced the apparent solid-phase retention capacity of Pb^2+^, while the overall transport regime remained relatively stable under the tested conditions. The limited variation in K_d_ among treatments further indicates that Pb^2+^ mobility was comparatively less sensitive to biochar-induced changes in water redistribution, consistent with the generally stronger retention tendency of Pb^2+^ in soil systems [7,8]. However, the linear representation should be interpreted as an adequate statistical description rather than direct evidence of completely equilibrium-controlled or irreversible Pb^2+^ sorption.

In contrast, Cd^2+^ transport exhibited stronger sensitivity to biochar and soil–water conditions. The partitioning-related parameter derived from the nonlinear model showed no significant difference between CK (0.17) and BACS1 (0.12) considering their overlapping uncertainty ranges, whereas BACS5 exhibited a marked increase (0.26), suggesting a stronger response at the higher biochar level. More importantly, the soil–water ratio threshold θ/ρ displayed clear treatment-dependent variation, shifting from 2.09 (CK) to 2.99 (BACS1) and 1.84 (BACS5). This variation suggests that biochar may influence Cd^2+^ redistribution not only through apparent sorption changes but also by modifying the relationship between water distribution and solute availability during unsaturated infiltration. Such hydrological effects are consistent with previous findings that biochar can alter soil hydraulic properties, including water retention and hydraulic conductivity [14]. Nevertheless, the threshold parameter represents an empirical descriptor derived from the observed transport behavior and should not be interpreted as a direct measure of a specific hydraulic process.

Integrating adsorption kinetics, isotherms, desorption behavior, and transport modeling, Pb^2+^ and Cd^2+^ exhibited contrasting transport characteristics under the tested conditions. Pb^2+^ showed relatively rapid adsorption, stronger apparent retention, and lower desorption reversibility, resulting in transport behavior that was well represented by linear partitioning. In comparison, Cd^2+^ exhibited weaker retention and greater sensitivity to water-dependent redistribution, requiring a nonlinear empirical model to better describe its variability. From an environmental perspective, these results suggest that biochar performance under unsaturated conditions depends not only on its ability to increase adsorption capacity but also on its influence on water-related transport processes, as water flow and soil–water distribution are known to drive solute redistribution during infiltration [32]. Therefore, biochar application strategies should consider metal-specific behavior and soil hydraulic conditions rather than relying solely on equilibrium sorption measurements [12].

## 4. Conclusions

This study investigated the effects of biochar on Pb^2+^ and Cd^2+^ adsorption, desorption, and transport behavior under transient unsaturated conditions by integrating batch adsorption experiments, column experiments under unsaturated conditions, and transport modeling. The results demonstrate that biochar modified the apparent retention behavior of both metals, but the magnitude and transport response were strongly element-dependent. Biochar addition increased the apparent adsorption capacity of Pb^2+^ and Cd^2+^, with a stronger response observed for Pb^2+^. It is worth noting that the biochar used in this study had a relatively limited specific surface area (12.83 m^2^/g), yet it still exhibited considerable adsorption capacity for Pb^2+^ and Cd^2+^. This suggests that, in the present system, the surface functional groups and surface chemistry of the biochar may contribute more to potentially toxic element adsorption than the specific surface area per se. According to previous studies, even with a low specific surface area, the effect of functional groups remains predominant in the material’s performance [33]. Kinetic and isotherm analyses indicated that adsorption behavior was better described by heterogeneous empirical models rather than a single-rate adsorption assumption, suggesting that biochar altered the apparent sorption environment of potentially toxic elements under the tested conditions.

The desorption experiments further revealed contrasting stabilization responses between Pb^2+^ and Cd^2+^. Biochar substantially reduced the apparent release of Pb^2+^, with BACS5 decreasing Pb^2+^ desorption by approximately 67.1% compared with the contaminated soil treatment, whereas Cd^2+^ exhibited relatively limited variation among treatments. These results indicate that biochar-induced immobilization was metal-specific, with Pb^2+^ showing stronger apparent retention and lower desorption reversibility than Cd^2+^. However, the observed differences represent macroscopic adsorption–desorption behavior, and the specific contributions of surface complexation, ion exchange, mineral interactions, or precipitation processes require further investigation using complementary spectroscopic and mineralogical techniques.

Under transient unsaturated infiltration conditions, Pb^2+^ and Cd^2+^ exhibited distinct transport behaviors. Pb^2+^ redistribution was adequately described by a linear partitioning relationship, indicating relatively stable solid–liquid distribution within the investigated experimental range. In contrast, Cd^2+^ showed stronger sensitivity to soil–water conditions, and the empirical nonlinear water-dependent model provided improved statistical representation of its redistribution behavior. The treatment-dependent variation of the soil–water ratio threshold θ/ρ suggests that biochar may influence Cd^2+^ transport not only through changes in apparent sorption capacity but also by modifying the relationship between water distribution and solute availability during unsaturated infiltration. Nevertheless, this nonlinear relationship should be interpreted as an empirical description of observed variability rather than direct evidence of a specific pore-scale transport mechanism.

Overall, the results of this study demonstrate that the effectiveness of biochar-based remediation under unsaturated conditions cannot be evaluated solely on the basis of equilibrium adsorption capacity; rather, potentially toxic element stabilization is governed by the combined effects of metal-specific retention characteristics and water-dependent transport variability. Pb^2+^ behavior was primarily characterized by partitioning-dominated retention, whereas Cd^2+^ exhibited greater sensitivity to soil–water redistribution. These findings indicate that hydrological conditions must be considered when evaluating the long-term performance of biochar in contaminated soils. It should be noted that the present conclusions are based on a homogeneously packed column system, and their extrapolation remains limited by the idealized experimental conditions pending field-scale validation. From an application perspective, the results of this study provide a basis for environmental risk and remediation assessment: groundwater contamination risk assessments for metal-contaminated soils should account for vadose-zone hydrological processes rather than relying solely on equilibrium adsorption parameters, and the design of biochar application schemes and the long-term evaluation of remediation effectiveness should also consider metal speciation, soil hydraulic conditions, and transport behavior to develop more targeted risk management and remediation strategies. Future studies integrating pore-scale structural characterization, long-term biochar aging, and field-scale reactive transport modeling are needed to further validate the persistence and applicability of biochar-based remediation strategies under heterogeneous environmental conditions.

## Figures and Tables

**Table 1:** List of chemical reagents employed in this study.

Reagent	Chemical Formula	Purity Grade	Manufacturer	City	Country
lead nitrate	Pb(NO_3_)_2_	analytical grade	Shanghai Aladdin Biochemical Technology Co., Ltd.	Shanghai	China
cadmium nitrate tetrahydrate	Cd(NO_3_)_2_·4H_2_O	analytical grade	Shanghai Aladdin Biochemical Technology Co., Ltd.	Shanghai	China
dilute nitric acid	HNO_3_	analytical grade	Chengdu Kelong Chemical Reagent Factory	Chengdu	China
hydroxylamine hydrochloride	NH_2_OH·HCl or HONH_3_Cl	analytical grade	Chengdu Kelong Chemical Reagent Factory	Chengdu	China
acetic acid	CH_3_COOH	analytical grade	Shanghai Aladdin Biochemical Technology Co., Ltd.	Shanghai	China
ammonium acetate	CH_3_COONH_4_	analytical grade	Shanghai Aladdin Biochemical Technology Co., Ltd.	Shanghai	China
sodium hydroxide	NaOH	analytical grade	Chengdu Kelong Chemical Reagent Factory	Chengdu	China
diethylenetriaminepentaacetic acid (DTPA)	C_14_H_23_N_3_O_10_	analytical grade	Tianjin Guangfu Fine Chemical Research Institute	Tianjin	China
triethanolamine	C_6_H_15_NO_3_	analytical grade	Tianjin Beichen Founder Reagent Factory	Tianjin	China
anhydrous calcium chloride	CaCl_2_	analytical grade	Shanghai Aladdin Biochemical Technology Co., Ltd.	Shanghai	China

**Table 2:** Adsorption kinetic model parameters of potentially toxic elements Cd^2+^ and Pb^2+^.

Tested Soil		CK		BAS1		BAS5	
		Pb^2+^	Cd^2+^	Pb^2+^	Cd^2+^	Pb^2+^	Cd^2+^
Pseudo-second-order kinetic model	K_2_ (×10^−3^) (kg/(mg⋅min))	2.34 ± 0.60	289.00 ± 96.06	2.58 ± 0.97	212.00 ± 55.20	2.06 ± 0.81	249.70 ± 101.47
	q_e_ (mg/kg)	4706.99 ± 7.99	98.06 ± 0.09	4717.09 ± 10.80	98.14 ± 0.09	4749.39 ± 14.09	98.22 ± 0.12
	R^2^	0.764	0.650	0.594	0.752	0.574	0.554
Elovich model	A (mg/(kg·min))	4612.45 ± 8.12	97.24 ± 0.10	4621.95 ± 9.22	97.10 ± 0.12	4628.00 ± 10.51	97.19 ± 0.10
	B (kg/mg)	14.01 ± 1.32	0.12 ± 0.02	14.35 ± 1.50	0.15 ± 0.02	18.26 ± 1.71	0.16 ± 0.02
	R^2^	0.958	0.922	0.948	0.922	0.959	0.952
Two-constant model	K_s_ (×10^−3^) (dimensionless)	2.99 ± 0.29	1.15 ± 0.20	3.06 ± 0.32	1.50 ± 0.23	3.89 ± 0.36	1.51 ± 0.09
	a (×10^−3^) (dimensionless)	8436.63 ± 1.73	4577.99 ± 1.39	8438.68 ± 1.94	4575.88 ± 1.40	8440.02 ± 2.17	4577.35 ± 0.31
	R^2^	0.956	0.871	0.948	0.897	0.960	0.983
Intra-particle diffusion model	K (mg/(kg·min^0.5^))	1.18 ± 0.33	0.01 ± 0.00	1.27 ± 0.33	0.01 ± 0.00	1.82 ± 0.33	0.02 ± 0.00
	C (mg/kg)	4658.71 ± 11.95	97.61 ± 0.11	4667.59 ± 11.95	97.58 ± 0.11	4681.11 ± 11.95	97.65 ± 0.11
	R^2^	0.667	0.778	0.728	0.747	0.914	0.877

**Table 3:** Isothermal adsorption model parameters of potentially toxic elements Cd^2+^ and Pb^2+^.

Tested Soil		CK		BAS1		BAS5	
		Pb^2+^	Cd^2+^	Pb^2+^	Cd^2+^	Pb^2+^	Cd^2+^
Henry model	K_H_ (L/kg)	318.77 ± 56.76	470.50 ± 30.96	323.41 ± 65.25	548.89 ± 27.70	406.81 ± 92.39	599.45 ± 25.70
	R^2^	0.840	0.975	0.804	0.985	0.764	0.989
Freundlich model	K_f_ (mg/kg)·(L/mg)^1/n^	2848.15 ± 111.74	728.18 ± 152.96	2088.82 ± 70.38	413.78 ± 77.95	2339.15 ± 87.29	496.39 ± 111.60
	1/n (dimensionless)	0.18 ± 0.02	1.21 ± 0.10	0.28 ± 0.01	0.84 ± 0.10	0.24 ± 0.02	0.90 ± 0.12
	R^2^	0.960	0.951	0.989	0.949	0.981	0.949

**Table 4:** Parameter estimation and performance evaluation of linear and nonlinear fitting models for Pb^2+^ and Cd^2+^ transport in soil columns under unsaturated conditions.

Model	Cd^2+^			Pb^2+^		
	CK	BACS1	BACS5	CK	BACS1	BACS5
Linear						
K_d_ (L/kg)	1.10 ± 0.37	1.25 ± 0.53	2.34 ± 0.70	1.43 ± 0.08	1.68 ± 0.03	1.54 ± 0.06
C_in_ (mg/L)	1.67 ± 0.56	1.50 ± 0.63	0.80 ± 0.24	240.91 ± 13.06	203.23 ± 3.10	215.39 ± 8.52
Q_in_ (mg/kg)	1.83 ± 0.08	1.88 ± 0.06	1.87 ± 0.03	345.03 ± 1.85	341.68 ± 0.30	333.47 ± 1.08
R^2^	0.689	0.533	0.652	0.988	0.998	0.990
RMSE	1.333	3.053	4.833	1.69	1.44	0.33
AICc	11.44	22.63	31.59	14.26	12.19	−8.35
Nonlinear						
K_d_ (L/kg)	0.17 ± 0.04	0.12 ± 0.01	0.26 ± 0.03	3.76 ± 94.45	4.88 ± 70.30	7.32 ± 53.34
w (mg^2^/L^2^)	9.80 ± 2.03	13.91 ± 1.27	6.75 ± 0.71	91.90 ± 2310.74	68.41 ± 986.40	46.67 ± 340.07
S (mg/L)	−20.49 ± 7.21	−41.52 ± 6.08	−12.43 ± 2.26	37.44 ± 307.50	28.89 ± 205.06	20.86 ± 102.99
θ/ρ*	2.09 ± 0.85	2.99 ± 0.51	1.84 ± 0.39	−0.41 ± 10.77	−0.42 ± 6.71	−0.45 ± 3.96
R^2^	0.916	0.963	0.951	0.989	0.992	0.999
RMSE	0.0376	0.0190	0.0151	1.60	1.37	0.32
AICc	−21.37	−41.49	−55.05	23.68	17.08	−1.88

## Data Availability

The raw data supporting the conclusions of this article will be made available by the authors on request.
